# Preoperative Stereotactic Radiosurgery for Glioblastoma

**DOI:** 10.3390/biology11020194

**Published:** 2022-01-26

**Authors:** Eric J. Lehrer, Henry Ruiz-Garcia, Anthony D. Nehlsen, Kunal K. Sindhu, Rachel Sarabia Estrada, Gerben R. Borst, Jason P. Sheehan, Alfredo Quinones-Hinojosa, Daniel M. Trifiletti

**Affiliations:** 1 Department of Radiation Oncology, Icahn School of Medicine at Mount Sinai, New York, NY 10029, USA; eric.lehrer@mountsinai.org (E.J.L.); anthony.nehlsen@mountsinai.org (A.D.N.); kunal.Sindhu@mountsinai.org (K.K.S.); 2 Department of Radiation Oncology, Mayo Clinic, Jacksonville, FL 32224, USA; ruizgarcia.henry@mayo.edu (H.R.-G.); estrada.rachel@mayo.edu (R.S.E.); 3 Department of Neurological Surgery, Mayo Clinic, Jacksonville, FL 32224, USA; Quinones-Hinojosa.Alfredo@mayo.edu; 4 The Christie NHS Foundation Trust, Wilmslow Road, Manchester M20 4BX, UK; gerben.borst@nhs.net; 5 Division of Cancer Sciences, School of Medical Sciences, Faculty of Biology, Medicine & Health, The University of Manchester, 555 Wilmslow Road, Manchester M20 4GJ, UK; 6 Department of Neurological Surgery, University of Virginia, Charlottesville, VA 22908, USA; jsheehan@virginia.edu

**Keywords:** glioblastoma, stereotactic radiosurgery, neurosurgery, radiation oncology, neuro-oncology, chemotherapy, radiation therapy, temozolomide, tumor treating fields, anti-tumor immunity

## Abstract

**Simple Summary:**

Stereotactic radiosurgery (SRS) is a multidisciplinary neurosurgical and radiation technique that allows for the delivery of a highly conformal dose of ionizing radiation with minimal radiation exposure to surrounding healthy tissues. SRS has been shown to be associated with excellent rates of local tumor control for multiple tumor types. Additionally, SRS has been shown to augment the effects of anti-tumor immunity. However, there is a paucity of evidence exploring the role of preoperative SRS in glioblastoma (GBM). To date, limited preclinical evidence has suggested that preoperative SRS has the potential to enhance anti-tumor immune responses and improve patient outcomes in glioma. In this review, we provide an overview of GBM and the role of preoperative radiosurgery in its management.

**Abstract:**

Glioblastoma is a devastating primary brain tumor with a median overall survival of approximately 15 months despite the use of optimal modern therapy. While GBM has been studied for decades, modern therapies have allowed for a reduction in treatment-related toxicities, while the prognosis has largely been unchanged. Adjuvant stereotactic radiosurgery (SRS) was previously studied in GBM; however, the results were disappointing. SRS is a highly conformal radiation technique that permits the delivery of high doses of ionizing radiation in 1–5 sessions while largely sparing surrounding healthy tissues. Furthermore, studies have shown that the delivery of ablative doses of ionizing radiation within the central nervous system is associated with enhanced anti-tumor immunity. While SRS is commonly used in the definitive and adjuvant settings for other CNS malignancies, its role in the preoperative setting has become a topic of great interest due to the potential for reduced treatment volumes due to the treatment of an intact tumor, and a lower risk of nodular leptomeningeal disease and radiation necrosis. While early reports of SRS in the adjuvant setting for glioblastoma were disappointing, its role in the preoperative setting and its impact on the anti-tumor adaptive immune response is largely unknown. In this review, we provide an overview of GBM, discuss the potential role of preoperative SRS, and discuss the possible immunogenic effects of this therapy.

## 1. Introduction

Gliomas are primary tumors of the brain that arise from astrocytes, ependymal cells, and oligodendrocytes [1]. Glioblastoma (GBM), the most common primary malignant tumor of the brain, accounts for approximately 50% of primary brain tumor diagnoses in the United States [2,3]. The peak incidence of GBM is seen in elderly patients and is more commonly observed in males than females [3].

GBM are highly aggressive tumors with a grim prognosis that are markedly resistant to treatment [4,5]. In the setting of optimal treatment, the median overall survival (OS) for patients with GBM ranges from 15–21 months with a 5-year OS rate of <5% [6,7,8,9,10]. Despite advances in surgical techniques, radiotherapy, and chemotherapeutic strategies in recent years, and the advent of tumor treating fields (TTF), outcomes for patients with GBM generally remain poor [6,7,8,9]. Stereotactic radiosurgery (SRS) in the adjuvant setting was previously studied in a randomized setting for GBM; however, the results were disappointing [11].

Stereotactic radiosurgery (SRS) is a radiation technique that allows for the delivery of a highly conformal dose of ionizing radiation with a rapid dose gradient outside of the target allowing for nearby healthy tissues to be spared [12,13,14]. Stereotactic radiosurgery is widely utilized for both malignant and benign intracranial pathologies [15,16,17,18,19,20,21,22,23,24,25,26,27,28,29,30,31,32,33,34,35]. Furthermore, multiple studies in the setting of brain metastases have suggested that SRS is able to enhance anti-tumor immunity through changes in the tumor microenvironment and can further augment intracranial responses to immune checkpoint inhibitors [36,37,38,39]. However, the role of SRS in the management of GBM is controversial [11,40,41]. In recent years, there has been a greater focus on the role of preoperative SRS, particularly in the setting of brain metastases [42]. This is due to a lower risk of nodular leptomeningeal disease and smaller treatment volumes in the preoperative setting, with a resultant decrease in the risk of radiation necrosis and normal tissue toxicity. The use of preoperative SRS has been largely unexplored in the management of GBM. However, there are many potential advantages to utilizing preoperative SRS in GBM, such as increased dose delivery and resultant changes in the tumor microenvironment which may enhance anti-tumor immunity against GBM.

In the forthcoming sections we will discuss laboratory and early preclinical evidence suggesting preoperative SRS may improve outcomes in GBM. Furthermore, we will review laboratory data suggesting that administration of immune checkpoint inhibitors can further amplify these effects. Finally, we will share data from our laboratory demonstrating the immunogenic effects of preoperative SRS in GBM. We also provide an overview of epidemiology and current treatment principles.

## 2. Epidemiology and Classification

The incidence of GBM is known to increase with patient age, with the peak incidence observed in patients aged 75 to 84 years [3]. However, cumulatively, the number of patients in the pre-retirement age is largest [43]. Males are more commonly diagnosed than females (5.6 vs. 3.5 cases per 100,000). Additionally, non-Hispanic White adults are more commonly affected than non-Hispanic Black adults (5.1 vs. 2.5 per 100,000). An increased risk has also been observed in patients with higher socioeconomic status; however, this association has only been shown to be significant among White individuals [44].

**Figure 1:**
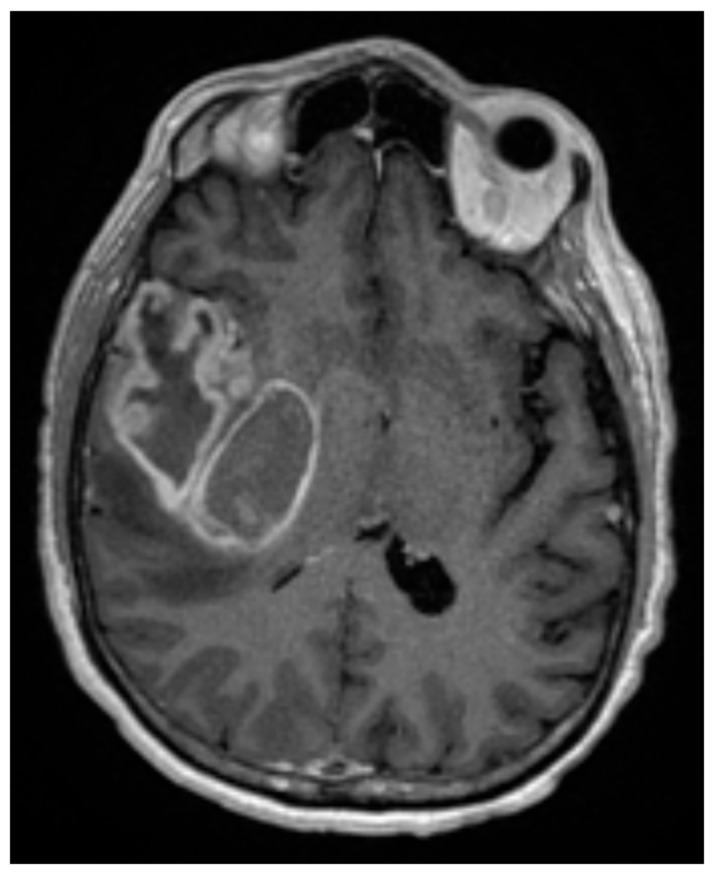
T1 post-contrast axial MRI of a 60-year-old man who presented with a 3-month history of progressively worsening headaches and memory loss. The MRI was consistent with a large contrast-enhancing mass in the right temporal lobe. He underwent a gross total resection, which demonstrated a 1p19q intact, IDH-wildtype, WHO grade 4 glioma, MGMT promoter unmethylated. Surgery was followed by concurrent external beam radiation therapy (60 Gy in 30 fractions) with concurrent TMZ, followed by adjuvant TMZ and TTF. He succumbed to his disease at 13 months following diagnosis.

The majority of GBM arise in the cerebral hemispheres [45], as shown in Figure 1. While several genetic syndromes, including (but not limited to) Li-Fraumeni Syndrome, Neurofibromatosis I and II, and Turcot Syndrome may predispose individuals to developing GBM, the only confirmed environmental risk factor is ionizing radiation [46,47].

Historically, the diagnosis of GBM was based on histological features alone. In general, GBM consist of small, polymorphic cells with an increased nuclear-to-cytoplasm ratio [48]. Binuclear and/or multinucleated giants cells may also be present [49]. However, the presence of microvascular proliferation and/or necrosis were required to truly distinguish grade 4 GBM from grade 2 and 3 gliomas [48,50].

In 2016, the World Health Organization (WHO) Classification of Tumors of the Central Nervous System for the first time incorporated molecular biomarkers into the classification of gliomas [51]. Under this system, all diffusely infiltrating gliomas, including astrocytomas, oligoastrocytomas, oligodendrogliomas, and GBM, were grouped together as diffuse gliomas. This category was then further subdivided by tumor classification and grade into 15 separate entities. Histologic GBM were themselves divided into three broad categories on the basis of isocitrate dehydrogenase (IDH) status: (1) glioblastoma, IDH-wildtype: most common in older patients, corresponded most commonly to primary GBM and encompassed approximately 90% of cases; (2) glioblastoma, IDH-mutant: most common in younger patients, corresponded most commonly to secondary GBM and encompassed approximately 10% of cases; and (3) glioblastoma, NOS, which was reserved for tumors in which full IDH testing could not be performed [50]. Patients with glioblastoma, IDH-mutant have better prognoses than those with glioblastoma, IDH-wildtype [52,53].

More recently, the 2021 WHO Classification of Tumors of the Central Nervous System has built upon the work enshrined by the WHO task force in 2016 [54]. Under the new framework, tumor grade has been eliminated as a classifier within the adult-type diffuse glioma family, thereby allowing for its further categorization into just three entities: astrocytoma, IDH-mutant; oligodendroglioma, IDH-mutant and 1p19q-codeleted; and glioblastoma, IDH-wildtype. Within the first two categories, the grade of the tumor may be used to further specify, but not classify, the tumor. As such, the glioblastoma, IDH-mutant classification has been eliminated and folded into the category astrocytoma, IDH-mutant, which includes grades 2–4 IDH-mutant astrocytomas. In addition, the process of grading tumors now also includes the evaluation of biomarkers rather than histologic analysis alone. Furthermore, the WHO has abandoned the use of Roman numerals and will now be utilizing Arabic numerals. In fact, CNS tumors may be classified as grade 4 in the absence of all characteristic histological changes as long as they possess a homozygous deletion of the gene *CDKN2A/B*. In addition, the inclusion criteria for a diagnosis of GBM, IDH-wildtype has expanded. In fact, “*Glioblastoma, IDH-wildtype should be diagnosed in the setting of an IDH-wildtype diffuse and astrocytic glioma in adults if there is microvascular proliferation or necrosis or TERT promoter mutation or EGFR gene amplification or +7/−10 chromosome copy number changes*” [54].

An important prognostic factor in GBM is the presence or absence of the methylation of the O^6^-methylguanine-DNA methyltransferase (MGMT) gene promotor. MGMT is a gene located on chromosome 10q26 that is responsible for removing alkyl groups from the O^6^ position of guanine, which is a common target of chemotherapeutic alkylating agents [55]. Patient OS has been shown to be prolonged in patients with MGMT gene promotor methylation who are treated with the alkylating agents temozolomide (TMZ) or carmustine (BCNU) [55,56]. Modern trials have demonstrated an approximate 6- month improvement in OS for patients with MGMT methylation treated with optimal therapy [6,7,55].

## 3. Treatment Overview

The optimal treatment paradigm for patients with GBM requires a multidisciplinary approach that typically involves maximal surgical resection followed by radiation therapy (RT) with concurrent and adjuvant TMZ chemotherapy and, finally, tumor-treating fields (TTF) [5,6,7,8,9]. However, treatment decision making is often complex and multiple factors must be considered, such as patient age and performance status, as certain populations are more likely to benefit from either intensified or de-intensified therapies [4,57,58,59,60]. This section provides an overview of current treatment strategies for GBM and describes how the available data influences the decision-making process.

### 3.1. Surgery

Maximal safe resection, while preserving neurological function, is the backbone of therapy for GBM [61]. Resection not only provides tissue for a definitive pathologic diagnosis but can also rapidly improve neurologic function through tumor debulking and decompression of mass effect [62]. The extent of resection (EOR) remains one of the most important prognosticators in patients with GBM, with survival being directly related to the degree of resection. Lacroix et al. were the first to describe this finding, demonstrating a 13-month median OS time in patients with ≥98% resection compared to just 8.8 months for patients with a less extensive surgery [63]. Additional studies have also shown that the EOR is associated with improved survival, even at resection thresholds as low as 70% [64]. However, the effect of the EOR on patient survival has been related to tumor molecular features. Beiko et al. demonstrated that the survival benefit differed based on IDH-status when resection of the non-enhancing tumor portion (supramaximal) was evaluated, being more prognostic in IDH-wild type diffuse gliomas [65]. Similar results were subsequently reported by Molinaro et al. [66].

This suggests that interventions aimed at enhancing the extent of resection and reducing the risk for patient injury play a critical role in improving outcomes in GBM [67,68]. For example, fluorescence-guided resection with 5-aminolevulinic acid has led to improved progression-free survival and gross total resection rates, and the use of intraoperative MRI has demonstrated improved outcomes too [69,70]. Other surgical techniques, such as awake craniotomy with cortico-subcortical mapping and neuronavigational systems also play a critical role in appropriately selected cases thereby preserving neurologic function and bettering patient outcomes [62,71,72]. Some have also advocated for a supratotal resection for GBM [73].

### 3.2. Systemic Therapy

Historically, nitrosourea-based chemotherapeutic agents were used in conjunction with RT as adjuvant therapy in the treatment of GBM, despite the lack of benefit demonstrated with these agents on phase 3 trials compared to RT alone [74,75,76]. This paradigm changed drastically in 2005 when a phase 3 randomized trial by Stupp et al. showed a significant improvement in median OS (from 12.1 to 14.6 months) with the addition of concurrent and adjuvant TMZ to radiation therapy and surgery [7]. This has now become standard of care therapy given the success of this trial and minimal treatment-related toxicity. Additionally, patients with a methylated MGMT gene promoter experienced median OS rates of 21.7 months [55].

Alternative chemotherapeutic agents, such as BCNU wafers, have also been shown to have some efficacy when placed at the time of surgery; however, it remains unclear whether they improve outcomes when delivered along with TMZ [77,78]. Unfortunately, other once-promising therapies, including bevacizumab, have not been shown to prolong survival when added to RT and TMZ, despite significant increases in progression-free survival [79,80].

### 3.3. Conventional Radiation Therapy

Despite the significant advancements in surgical techniques, GBM remains an aggressive disease with high rates of local failure. Even with aggressive therapy and extensive surgery, tumors recur within two centimeters of the primary site in up to 90% of patients [62]. Due to this, RT has been delivered to patients to delay tumor recurrence and improve survival outcomes [7]. In 1978, Walker et al. published the first randomized trial establishing the role of adjuvant chemoradiotherapy in the management of GBM [81]. Three hundred and three patients with anaplastic gliomas who underwent a resection were randomized to supportive care, BCNU chemotherapy alone, radiotherapy alone, or BCNU chemoradiotherapy. The trial demonstrated a median OS of 34.5 weeks vs. 18.5 weeks in the chemoradiotherapy and chemotherapy alone arms, respectively. 

In 2005, as discussed above, Stupp et al. published a seminal phase 3 randomized trial that established the role of adjuvant chemoradiotherapy in the setting of newly diagnosed GBM [7]. Patients in the chemoradiotherapy arm were treated to a dose of 60 Gy. The planning target volume (PTV) was defined as the gross tumor volume (GTV) with a 2–3 cm expansion accounting for areas at high risk for subclinical disease (CTV). Patients in the chemoradiotherapy arm experienced a longer median OS of 14.6 months vs. 12.1 months for the chemotherapy alone arm. 

Radiation target delineation in GBM remains a controversial topic [82]. The controversy involves whether to include the entirety of the FLAIR signal in the treatment volume. Proponents argue that incorporation of the FLAIR signal allows for the inclusion of visibly suspicious areas that may harbor malignant cells. While opponents argue that since the majority of GBM exhibit local failures within 2 cm of the resection cavity, increasing tumor volumes significantly beyond this point is associated with an unacceptable risk of treatment-related toxicity for a potentially minimal patient benefit [83]. Current target delineation guidelines for GBM are presented in Table 1. Interestingly, a 2014 study utilizing the Adult Brain Tumor Consortium (ABTC) guidelines by Gedhardt et al. noted that 81% of patient failures occurred within the RT treatment field [84]. This further supports the hypothesis that smaller treatment volumes may be equally efficacious.

**Figure 2:**
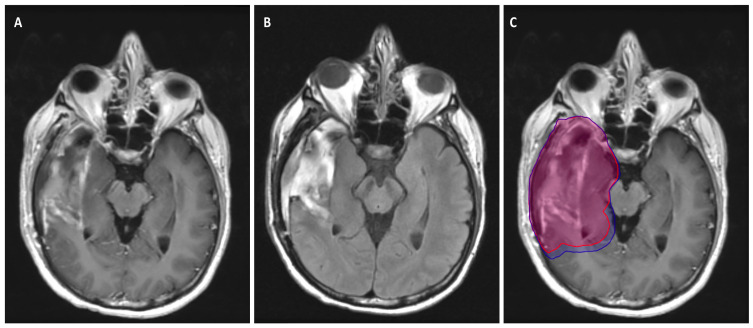
A patient who was diagnosed with a right temporal lobe GBM. He underwent gross total resection and was subsequently treated with 60 Gy in 30 fractions with concurrent TMZ, followed by adjuvant TMZ. The RTOG approach was used to generate the target volumes. (**A**) T1 post-contrast axial MRI taken 48 h post gross total resection with a large postoperative cavity in the right temporal lobe; (**B**) T2 FLAIR axial MRI at the same level as (**A**) demonstrating significant surrounding edema; (**C**) Planning scan with PTV1 and PTV2 overlaid on (**A**). The area encompassed by the blue volume is receiving 46 Gy in 23 fractions, while the area encompassed by the red volume is receiving an additional 14 Gy in 7 fractions to a total dose of 60 Gy in 30 fractions.

Figure 2 depicts a patient with a GBM who was treated using the Radiation Therapy Oncology Group (RTOG) target delineation guidelines.

Dose escalation beyond 60 Gy remains a controversial topic in the management of GBM [4]. The only prospective evidence comparing standard of care chemoradiotherapy to dose-escalated radiotherapy is NRG BN-001. Preliminary results presented at the 2020 meeting for the American Society of Radiation Oncology demonstrated no statistically significant improvement in median OS with the addition of dose-escalated radiotherapy without a significant increase in grade 3 + toxicity. However, it is important to note that these results were limited to the photon arm of the study, and both photons and protons are permitted radiation modalities on this trial.

### 3.4. Tumor-Treating Fields

Tumor-treating fields, which consist of alternating, low-intensity electric fields applied to the scalp, have also proven to be beneficial in patients with GBM [8,9]. When added to adjuvant RT and TMZ, Stupp et al. reported an OS improvement from 16 months to 20.9 months [8,9]. Additionally, the treatment was well-tolerated, with only mild to moderate skin toxicities noted at an increased frequency in the TTF group. Given the favorable survival and safety data, TTF has also been established as a standard of care therapy for patients with GBM. However, given the lack of knowledge about its mechanism of action, critiques regarding the trial design, and financial concerns, there remains some degree of skepticism in the neuro-oncology community regarding the routine use of TTF [85].

### 3.5. Treatment Considerations in the Elderly Population

Due to the high prevalence of GBM in older populations and the poorer prognosis in many of these patients, numerous efforts have attempted to optimize outcomes by balancing therapeutic interventions with associated toxicities [57,58,59,60,86,87]. Surgery has been shown to improve OS in cohorts of elderly patients and is frequently recommended, as patient functional status allows [88]. While RT has proven to be significantly more effective than supportive care alone, numerous studies have shown that de-intensified treatment strategies result in comparable outcomes compared to standard of care therapies. A study by Roa et al. demonstrated the equivalence of a hypofractionated course of RT (40 Gy in 15 fractions) with the standard 60 Gy in 30 fractions in patients greater than 60 years old, with a median OS of 5.1 months vs. 5.6 months (*p* = 0.57) for the hypofractionated and standard RT arms, respectively [58]. Furthermore, a subsequent study by Roa et al. in elderly or frail patients compared hypofractionated RT (40 Gy in 15 fractions) to short-course RT (25 Gy in 5 fractions [59]. They observed no differences in OS, progression-free survival, or quality of life between the two treatment regimens. 

The incorporation of TMZ has also been evaluated in the elderly population. The NOA-08 trial, which compared 60 Gy to 40 Gy or TMZ alone found that 60 Gy was associated with especially poor outcomes. Additionally, patients with MGMT promotor methylation who received TMZ alone had a superior event-free survival when compared to those who received RT, while the opposite was true for patients who were MGMT unmethylated [87]. Similar results were observed in the Nordic trial published by Malmström et al. in 2012, which concluded that hypofractionated RT and TMZ should be considered as treatment options in the elderly population and that MGMT methylation status is a valuable prognostic indicator for response to TMZ [60]. More recently, Perry et al. sought to determine whether chemotherapy should be added to hypofractionated RT. They found that not only was the addition of TMZ well-tolerated, but TMZ also improved OS from 7.7 to 13.5 months [57].

## 4. Stereotactic Radiosurgery

### 4.1. Overview

Stereotactic radiosurgery was first proposed in 1951 by Dr. Lars Leksell at the Karolinska Institute in Stockholm, Sweden [12]. It allows for the delivery of an ablative dose of RT in a single session with minimal dose delivery to surrounding healthy tissues. While SRS was originally performed in a single session, fractionated SRS or fractionated stereotactic radiotherapy uses the same treatment principles over 1–5 sessions [25,89,90]. This fractionated treatment strategy is commonly utilized for larger lesions and/or lesions in eloquent locations, such as the brainstem [25,33,34,89,90,91]. Major advantages of SRS include markedly shortened treatment times compared to conventionally fractionated radiotherapy and a lower risk of treatment-related toxicity due to lower radiation dose delivery to nearby healthy tissues. This is best illustrated in the treatment of limited brain metastases. Traditional approaches, such as whole brain radiation therapy (WBRT), while associated with excellent rates of tumor control, require 3–4 weeks of treatment and are associated with a significant risk of cognitive sequelae [17,92,93,94,95]. SRS has demonstrated excellent rates of tumor control with markedly decreased rates of cognitive sequelae and is now widely used in this setting [15,16,17,96]. In recent years, there has been heightened interest in utilizing preoperative SRS in the management of brain metastases, as it is associated with decreased rates of radiation necrosis and nodular leptomeningeal disease, while allowing for smaller treatment volumes when compared to adjuvant SRS delivered to the postoperative tumor bed [42].

### 4.2. Stereotactic Radiosurgery in Glioblastoma in the Adjuvant Setting

Due to the poor prognosis of GBM and studies demonstrating that majority of recurrences occur with 2 cm of the resection cavity, there has been interest in dose escalation while minimizing further exposure to nearby healthy tissues [40,41,83]. The highest quality evidence evaluating the role of SRS in GBM is the RTOG 9305 randomized trial [11]. This trial randomized 203 patients with supratentorial GBM measuring no more than 40 mm to 60 Gy in 30 fractions and BCNU with or without the addition of postoperative SRS. Radiosurgery dosing was based on dose recommendations from RTOG 9005 [97]. At a median follow-up time of 61 months, the median OS was 13.5 months vs. 13.6 months for the SRS and non-SRS groups, respectively. While these results failed to show a benefit to SRS, they are not reflective of current standard treatment practices, such as the use of concurrent and adjuvant TMZ [7]. Additionally, the impact of SRS sequencing in the management of GBM remains largely unknown [5].

### 4.3. Rationale for Preoperative Stereotactic Radiosurgery in Glioblastoma

Utilization of SRS in the preoperative setting provides multiple advantages compared to the postoperative setting. First, preoperative therapy allows for smaller RT target volumes and more precise target delineation, thus decreasing dose delivery to nearby healthy tissues and lowering the risk of treatment-related toxicities (e.g., radiation necrosis). Second, intact tissues have higher oxygen concentrations, which allow for more effective RT-induced DNA double-stranded breaks either directly or indirectly via the action of reactive oxygen species [98]. Third, post-irradiation tissue analysis is possible with preoperative therapy, which would permit further characterization of repair pathways and tumor microenvironment changes following SRS. This has the potential to allow for more individualized treatment strategies for these patients. Additionally, characterization of these pathways can provide an opportunity for future research into novel therapeutics targeting specific molecular targets following preoperative SRS. Fourth, the risk of nodular leptomeningeal disease for brain metastasis patients appears lower when SRS is administered preoperatively [42]. The risk of leptomeningeal disease for GBM patients is approximately 4% and frequently represents a dismal development for GBM patients [99,100].

## 5. Immunogenic Effects of Ionizing Radiation

Preclinical models have demonstrated that RT has the potential to act as an in-situ tumor vaccine due to an associated increase in the release of tumor-associated antigens, which allows for CD8^+^ T-cell priming and the generation of an adaptive immune response [101]. It has also been demonstrated that ablative doses of radiation induce multiple mechanisms that increase antigen presentation and CD8^+^ T cell activation [36,37,101,102,103,104,105]. This synergy is a major area of current investigation, particularly when used in combination with immune checkpoint inhibitors (ICI) in the management of brain metastases [36,37,38,106,107,108,109,110].

Studies have also demonstrated that ionizing radiation alters the tumor microenvironment and enhances anti-tumor immunity in gliomas [111,112]. In 1994, Klein et al. published a study in which GBM specimens were exposed to escalating doses of RT [111]. They observed RT was associated with an increase in expression of major histocompatibility complex (MHC) class I antigen expression when compared with controls, thus suggesting that RT may enhance cytotoxic T-cell activity against GBM. A subsequent study by Newcomb et al. evaluated the impact of WBRT combined with vaccination in mouse GL261 gliomas [112]. While WBRT or vaccination alone had a minimal impact on survival, the combination of these two therapies increased long-term survival to 40–80%. Additionally, WBRT was associated with increased expression of MHC class I and *β2*-microglobulin, as well as an increased concentration of infiltrating lymphocytes within the tumor. However, given that WBRT is not a standard treatment in GBM, these data need to be validated in more clinically relevant models. In 2012, Zeng et al. published a study that evaluated mouse models of GBM that were treated with anti-PD-1 therapy and SRS to a dose of 10 Gy [113]. This study found a median OS of 25, 27, 28, and 53 days in the control, anti-PD-1 only, SRS only, and SRS + anti-PD-1 arms. These findings suggest that RT enhances anti-tumor immunity against glioma cells, which may be further amplified by ICI. 

**Figure 3:**
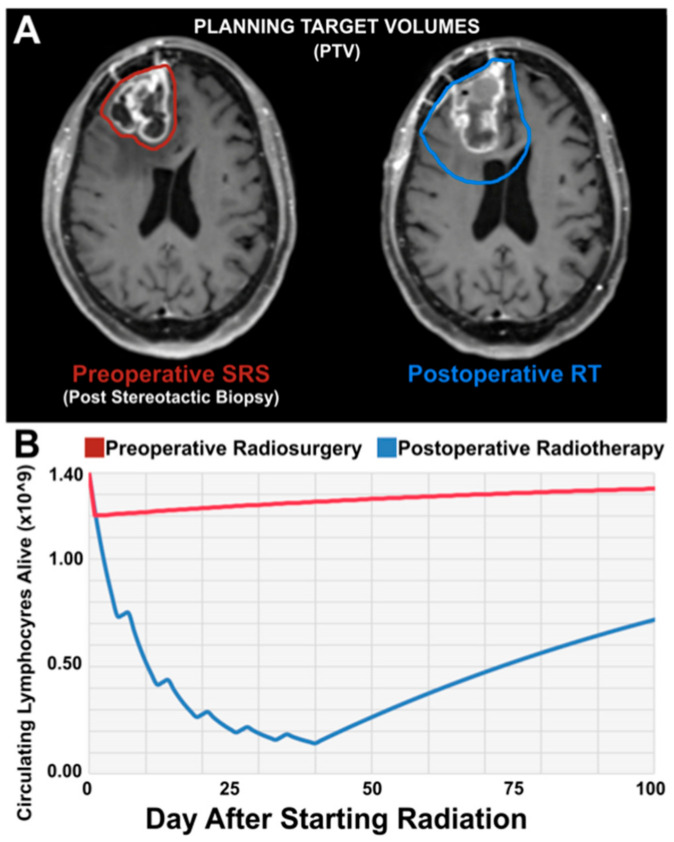
Preoperative SRS irradiates less healthy brain and kills less lymphocytes compared to Postoperative RT. (**A**) Preoperative (left) and postoperative (right) MRI showing preoperative SRS PTV (red) as compared to standard postoperative RT PTV (blue). As demonstrated, the vast majority of tissue irradiated during preoperative SRS is resected and minimal dose of radiation is delivered by SRS beyond the tumor bulk. (**B**) Changes in serum naive CD8+ lymphocytes after SRS compared to postoperative RT based on our preliminary mathematical modeling. Staggered edge reflects weekend recovery.

Due to the advantages of preoperative compared to adjuvant SRS and the strong preclinical evidence suggesting that SRS enhances anti-tumor immunity against GBM cells, this therapy represents an opportunity to improve patient outcomes. Figure 3 depicts data from our laboratory. Mathematical modeling was used to calculate changes in CD8^+^ T cells following preoperative SRS and postoperative SRS. As shown, the CD8^+^ T cell population is markedly higher in the preoperative setting, caused by differences in irradiation volume, dose, and frequency with preoperative SRS. This would suggest that preoperative SRS has the potential to enhance anti-tumor immunity in this setting.

## 6. Treatment Considerations in Preoperative Radiosurgery

When delivering preoperative SRS, the GTV should be contoured using a T1 contrast enhanced sequence and should encompass all enhancing disease. The use of a PTV margin is at the discretion of the treating provider. On the NeoGlioma Study (NCT05030298), which is investigating the role of preoperative SRS in high-grade glioma, a PTV margin of 3 mm was recommended due to the use of linear accelerator SRS. However, clinicians may employ a margin of 0–3 mm. A CTV margin is not utilized in this setting. Once the patient undergoes resection, the use of a CTV to address subclinical disease due to the diffuse and infiltrative nature of GBM is recommended.

The timing of subsequent surgical resection is also an important consideration, particularly to maximize anti-tumor immunity. While there are limited data studying this specific scenario. A preclinical study by De La Maza et al. utilized a mesothelioma murine model and treatment with hypofractionated RT [114]. They observed that surgery alone or treatment with surgery followed by hypofractionated RT after 1 day resulted in no tumor rejection when a re-challenge was performed at 90 days. However, when surgery was performed 7 days following RT, a 90-day re-challenge resulted in a 33% complete tumor rejection. Additionally, this cohort had the lowest growth rate overall. The investigators noted that this was an immunologic finding, as CD4+ T-cell depletion markedly diminished these findings. This suggests that long-term immunologic memory against the tumor is improved when surgery does not immediately follow RT. On the NeoGlioma study, surgical resection is specified to occur within 14 days following SRS. However, clinical and logistical circumstances may make waiting this time period infeasible.

## 7. Conclusions

Glioblastoma is a devastating malignancy associated with a median OS of approximately 15 months, even when optimal modern therapy is utilized. While SRS trials in the adjuvant setting have been disappointing, its role in the preoperative setting in combination with modern systemic agents has been largely unexplored. Preclinical data suggest that preoperative SRS can further enhance immune responses against GBM, which may be leveraged to improve OS. Further studies are needed to better define the role of preoperative SRS in this setting. Additional preclinical and early clinical investigations are underway to further evaluate this therapy, such as the NeoGlioma study (NCT05030298) which is a phase 1/2A clinical trial evaluating the role of preoperative SRS in high-grade glioma.

## Figures and Tables

**Table 1:** Current GBM contouring guidelines by the European Organization for Research and Treatment of Cancer (EORTC), the Radiation Therapy Oncology Group (RTOG), and the Adult Brain Tumor Consortium (ABTC).

EORTC	RTOG	ABTC
Phase 1: 60 Gy in 30 fractions	Phase 1: 46 Gy in 23 fractions	Phase 1: 46 Gy in 23 fractions
GTV: resection cavity + residual T1 postcontrast enhancement	GTV_1_: resection cavity + residual T1 post-contrast enhancement + surrounding edema (FLAIR)	GTV_1_: T1 enhancing and non-enhancing tumor volume (T2 or FLAIR)
CTV: GTV + 2 cm	CTV_1_: GTV_1_ + 2 cm	CTV_1_: GTV_1_ + 5 mm
PTV: CTV + 3–5 mm	PTV_1_: CTV_1_ + 3–5 mm	PTV_1_: CTV_1_ + 3–5 mm
	**Phase 2: 14 Gy in 7 fractions**	**Phase 2: 14 Gy in 7 fractions**
	GTV_2_: resection cavity + residual T1 post-contrast enhancement	GTV_2_: T1 enhancing tumor volume
	CTV_2_: GTV_2_ + 2 cm	CTV_2_: GTV_2_ + 5 mm
	PTV_2_: CTV_2_ + 3–5 mm	PTV_2_: CTV_2_ + 3–5 mm

## Data Availability

Preliminary data presented in this study are available upon request to the corresponding author.
